# Efficacy and safety of mirabegron versus solifenacin in the treatment of overactive bladder in children: a systematic review and meta-analysis

**DOI:** 10.1186/s12894-026-02155-9

**Published:** 2026-04-18

**Authors:** Mostafa Shaheen, Omar Mohamed Ali, Abdullah A. Draz, Amro K. Barakat, Ahmed Elghattas, Toqa Elbaz, Iyad Walid, Tasnim Tarboush, Asmaa Elsayed, Tasneem Mahmoud, Esraa Mohamed Elkhateeb, Abdallah Adel Nofal, Emad Samaan

**Affiliations:** 1 Faculty of Medicine, Mansoura University, Mansoura, 35516 Egypt; 2 Department of Internal Medicine – Nephrology Unit, Mansoura University Hospitals, Mansoura, Egypt; 3 Faculty of Medicine, Mansoura Manchester Research Society (MMRS), Mansoura University, Mansoura, Egypt

**Keywords:** Children, Mirabegron, Overactive Bladder, Solifenacin

## Abstract

**Background:**

Overactive bladder (OAB) affects 5–12% of children and is associated with urgency, frequency, and incontinence, often leading to psychosocial distress. Antimuscarinics are widely used but limited by systemic side effects. Mirabegron, a β3-adrenoceptor agonist, may offer better tolerability. This review evaluated available evidence comparing mirabegron and solifenacin in pediatric OAB.

**Methods:**

Following PRISMA guidelines (PROSPERO: CRD420251129236), we searched PubMed, Scopus, Web of Science, and the Cochrane Library through September 2025 for studies directly comparing mirabegron and solifenacin in children ≤ 18 years with non-neurogenic OAB. Two reviewers independently screened studies, extracted data, and assessed quality. Risk ratios (RRs) with 95% confidence intervals (CIs) were pooled using random-effects models.

**Results:**

Three studies (246 children; 116 mirabegron, 130 solifenacin) met inclusion criteria. Pooled analysis showed no significant difference in treatment response between groups (RR = 1.11, 95% CI: 0.92–1.33; *p* = 0.28; I² = 58%). Mirabegron was associated with significantly fewer adverse events (RR = 0.44, 95% CI: 0.28–0.70; *p* = 0.0004; I² = 0%).

**Conclusions:**

Mirabegron and solifenacin demonstrate comparable efficacy in pediatric OAB, with mirabegron suggesting a more favorable short-term safety profile. These preliminary findings require confirmation in larger, long-term trials with standardized outcome measures.

**Clinical Trial Registration:**

Not applicable.

**Supplementary Information:**

The online version contains supplementary material available at 10.1186/s12894-026-02155-9.

## Introduction

Overactive bladder (OAB) represents one of the most prevalent and clinically significant pediatric urological conditions, affecting approximately 5–12% of children aged 5–10 years worldwide [1]. According to the International Children’s Continence Society, pediatric OAB is defined as urinary urgency, typically with frequency and nocturia, with or without urinary incontinence, occurring without urinary tract infection or other obvious pathology [2].

Pediatric OAB presents as a complex set of urinary symptoms that impact children and families [3]. Symptoms include increased daytime frequency, an urgent need to urinate with holding maneuvers, daytime accidents, and nocturnal enuresis [4]. In addition to physical symptoms, it also leads to psychosocial consequences, as children often face social embarrassment and isolation, withdraw from peer activities, and experience a lower quality of life that impacts both their development and family dynamics [5].

The management of pediatric OAB follows a stepwise approach, starting with conservative measures such as behavioral interventions and lifestyle modifications before pharmacological interventions [6]. When non-pharmacological methods fail, antimuscarinic agents are the primary medical treatment [7]. Despite their efficacy, they are limited by anticholinergic side effects such as dry mouth, constipation, blurred vision, and headache [8].

Mirabegron stimulates β3 receptors in the detrusor muscle, inducing relaxation during storage, which improves bladder capacity and decreases contractility [9]. In contrast to antimuscarinics, it maintains normal voiding function and reduces residual urine [10]. A comprehensive pooled analysis of three large phase III studies revealed that mirabegron significantly improved incontinence and micturition, with efficacy comparable to that of antimuscarinics and superior tolerability in adults with OAB [11].

Mirabegron has been shown in multiple trials to be effective in treating OAB in adults and was adopted by the NICE guidelines in 2013 [12–14]. As a β3 agonist, it also appears safe and effective in children with OAB, improving urodynamic parameters and clinical symptoms, and provides an alternative for refractory or antimuscarinic-intolerant patients [13, 15]. Previous studies comparing mirabegron and solifenacin in paediatric OAB patients have shown varying results. Soliman et al. reported that both agents are effective, with mirabegron showing better tolerability [16]. Kim et al. reported similar efficacy and safety, whereas Mansour et al. suggested that mirabegron may offer superior outcomes with fewer adverse effects [17, 18]. However, no systematic review has compared these agents in children. Therefore, this review aimed to evaluate evidence comparing the efficacy and safety of mirabegron versus solifenacin in children with OAB, aiming to offer insights that can guide clinical decisions and optimize treatment.

## Methods

### Protocol

This systematic review and meta-analysis were conducted and reported in accordance with the Preferred Reporting Items for Systematic Reviews and Meta-Analyses (PRISMA) guidelines [19]. The study protocol was registered with the International Prospective Register of Systematic Reviews (PROSPERO) under the registration number CRD420251129236.

### Search strategy

A comprehensive literature search was conducted across four electronic databases: PubMed, Scopus, Web of Science, and the Cochrane Library, up to September 2, 2025. No date restrictions were applied; however, the search was limited to studies published in English. The search strategy combined keywords and medical subject headings related to the pediatric population, overactive bladder, mirabegron, and solifenacin. The detailed search strategy for databases is provided in Appendix 1.

### Eligibility criteria

Studies eligible for this review included randomized controlled trials, clinical trials, prospective or retrospective cohort studies, and case‒control studies that directly compared mirabegron and solifenacin as monotherapies. The study population was children and adolescents (≤ 18 years) diagnosed with non-neurogenic OAB. Conversely, studies were excluded if they were case reports, case series, reviews, or editorials. Additionally, studies without a direct comparator arm or those focusing exclusively on neurogenic bladder populations were not included.

### Outcome definition

The efficacy outcome was treatment response, although its definition varied across the included studies. Mansour et al. defined efficacy as a ≥ 50% reduction in the baseline Dysfunctional Voiding Scoring System (DVSS) score [18]. Soliman et al. used a similar threshold, defining success as a > 50% improvement based on a symptom relief questionnaire [16] In contrast, Kim et al. defined a response as a ≥ 90% reduction in symptoms based on patient and parent reports [17]. The safety outcome was assessed as the occurrence of any treatment-related adverse events.

### Study selection

Studies imported into the Rayyan web application for systematic reviews [20]. After duplicate records were manually removed, two reviewers independently and blindly screened the titles and abstracts of the remaining articles to assess their relevance. The full texts of the studies that met our predetermined inclusion criteria were subsequently screened. Any disagreements during the selection process were resolved by discussion and agreement with a third reviewer. The flow diagram was generated via the PRISMA2020 R package and Shiny app [21].

### Quality assessment and publication bias

The quality of the included studies was independently assessed by two blinded reviewers, with any disagreements resolved by a third reviewer. Randomized con­trolled trials were evaluated with the Cochrane Risk of Bias tool 2 (RoB 2) [22] which covers five domains: the randomization process, deviations from intended interventions, missing outcome data, measurement of the outcome, and selection of the reported result. Each domain is rated as ‘low risk,’ ‘high risk,’ or ‘some concerns.’ Non-randomized studies, including cohort designs, were assessed using the Newcastle–Ottawa Scale (NOS) [23]. Three areas are evaluated: selection of study groups (up to four stars), comparability of groups (up to two stars), and ascertainment of exposure or outcome (up to three stars). Based on total scores, studies were categorized as high, moderate, or low quality, with higher scores indicating better methodological rigor.

### Statistical analysis

Dichotomous outcomes (treatment response and the incidence of adverse events) were analyzed using the Risk Ratio (RR) with its 95% confidence interval (CI) to estimate effects. Due to the expected clinical and methodological heterogeneity among the included studies, a random-effects model was employed for meta-analysis. Statistical heterogeneity was measured with Higgins’ I² test. According to established guidelines, an I² value of 0–40% indicates insignificant heterogeneity, 30–60% suggests moderate heterogeneity, 50–90% reflects substantial heterogeneity, and 75–100% shows considerable heterogeneity [24]. To test the robustness of the results and determine the influence of each study on the overall estimate, a sensitivity analysis was performed by removing one study at a time and reanalyzing the data. All statistical analyses for this review were conducted using Review Manager software Version 5.3 [25].

## Results

### Study selection

**Fig. 1:**
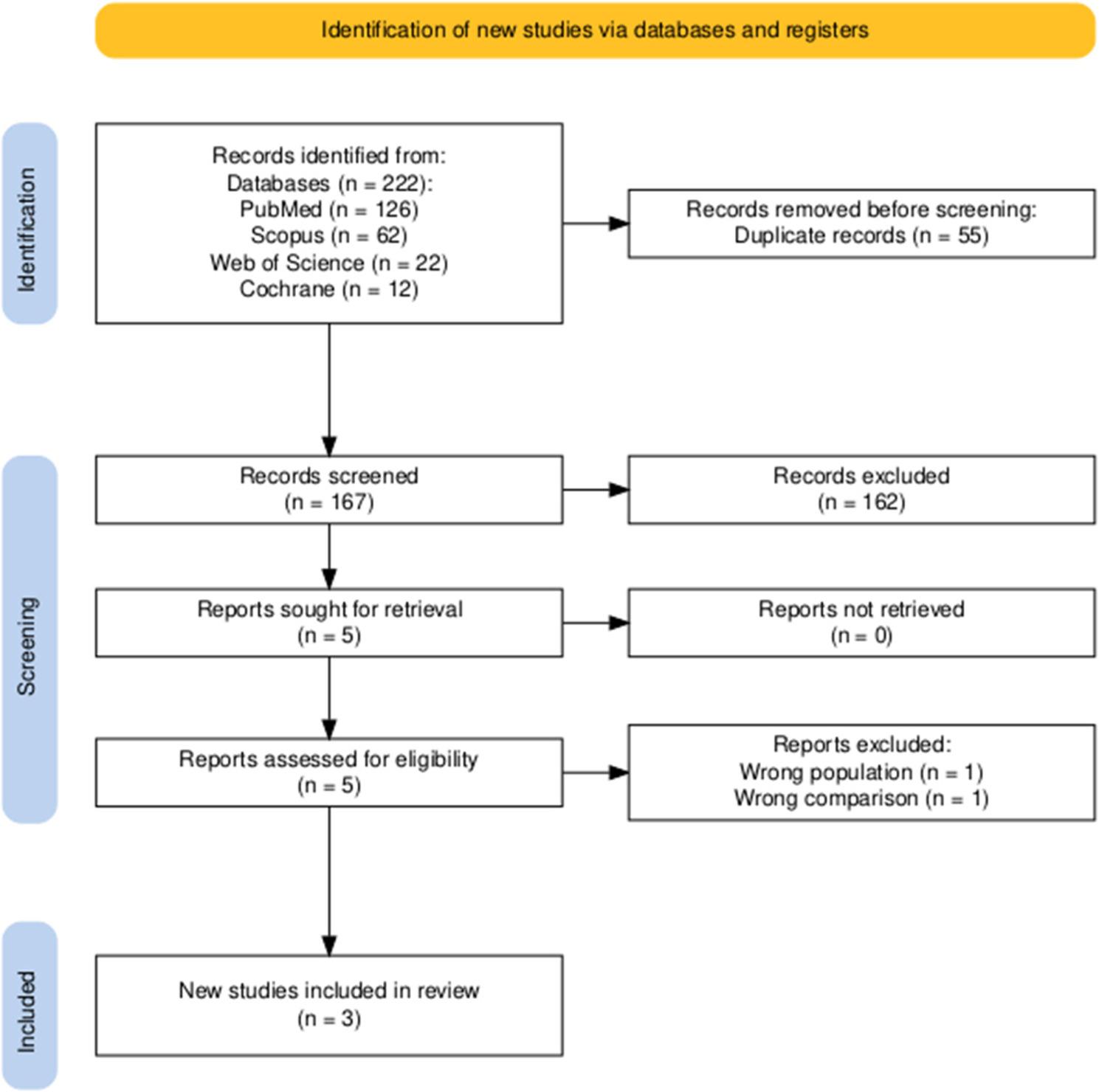
Prisma flow diagram of the included studies

As shown in Fig. 1, our search strategy yielded a total of 222 studies from four databases. After eliminating 55 duplicate records, 167 studies remained for screening. Of these, 162 were excluded based on title and abstract screening. After full-text screening, three studies were included in the review, involving a total of 246 patients, with 116 patients treated with mirabegron and 130 patients treated with solifenacin.

### Baseline characteristics

The total sample size across the included studies was 116 patients in the mirabegron group and 130 patients in the solifenacin group. The mean age (SD) was 9.44 years (1.97) for the Mirabegron group and 8.55 years (2.21) for the Solifenacin group, with an overall range of 5–15 years across included studies. Overall, 41.8% of the participants were male. The Mansour and Soliman studies followed both treatment groups for 12 weeks, whereas Kim et al. followed Mirabegron patients for 34 weeks and Solifenacin patients for 28 weeks. The baseline characteristics of the included studies are shown in Table 1.

**Table 1:** Baseline characteristics of included studies

Study ID	Group	Sample Size	Age in years (mean ± SD)	Sex (Male *n*, %)	Baseline Symptom (*n*, %)	Duration (weeks)
Kim et al., [17]	Mirabegron	16	9.70 ± 2.19^a^	10 (62.5%)	Daytime incontinence: 13/16 (81.3%); Nocturnal enuresis: 12/16 (75.0%)	34 weeks
	Solifenacin	29	6.43 ± 1.17^a^	12 (41.4%)	Daytime incontinence: 23/29 (79.3%); Nocturnal enuresis: 18/29 (62.1%)	28 weeks
Mansour et al., [18]	Mirabegron	36	9.4 ± 2.14	14 (39%)	Daytime incontinence: median 2 episodes/day (range 0–5); Nocturnal enuresis: 22/36 (61.1%)	12 weeks
	Solifenacin	36	9.1 ± 2.34	10 (28%)	Daytime incontinence: median 2 episodes/day (range 1–4); Nocturnal enuresis: 21/36 (58.3%)	12 weeks
Soliman et al., [16]	Mirabegron	64	9.4 ± 1.8	27 (42.2%)	Incontinence episodes/day: 2.7 ± 0.8	12 weeks
	Solifenacin	65	9.2 ± 1.9	30 (46.2%)	Incontinence episodes/day: 2.9 ± 0.9	12 weeks

SD = standard deviation; ^a^Median (IQR) converted to mean ± SD using Wan et al. method [26]

### Clinical characteristics and outcomes

Table 2 outlined the characteristics and clinical outcomes of pediatric patients with OAB treated with mirabegron versus solifenacin across three studies: Kim et al., Mansour et al., and Soliman et al. Mansour et al. and Soliman et al. were single-blind randomized controlled trials (RCTs), whereas Kim et al. was an observational study. All studies included pediatric patients with idiopathic non-neurogenic OAB aged from 5 to 15 years. The intervention consisted of a daily single dose of mirabegron at 50 mg, with weight-adjusted dosing in Mansour et al., whereas the comparator was a daily single dose of solifenacin at 5 mg across all studies. Fewer adverse events were consistently associated with mirabegron than with solifenacin. The efficacy outcomes showed that Mirabegron was comparable to solifenacin in Kim et al. and Soliman et al., whereas Mansour et al. reported greater efficacy for Mirabegron.

**Table 2:** Characteristics and clinical outcomes of included studies

Study ID	Study Design	Population	Intervention	Comparator	Efficacy Outcomes	Safety Outcomes	Key findings
Kim et al., [17]	Retrospective observational study	Pediatric patients aged 5–15 years with idiopathic OAB	Mirabegron 50 mg	Solifenacin 5 mg	Response rate (≥ 90% improvement in OAB symptoms): Solifenacin 20/29 (69.0%) vs. Mirabegron 13/16 (81.3%); *p* = 0.491	Solifenacin (*n* = 29): adverse events 3/29 (10.3%) – abdominal cramps (1), anorexia (1), nausea (1). Mirabegron (*n* = 16): 0/16 (0%)	Mirabegron showed similar efficacy to solifenacin with no observed adverse effects
Mansour et al., [18]	Single-blinded randomized controlled trial	Children aged 5–12 years with non-neurogenic OAB unresponsive to urotherapy	Mirabegron 25 mg once daily (< 40 kg); 50 mg once daily (≥ 40 kg)	Solifenacin 5 mg once daily	≥ 50% DVSS reduction: Mirabegron 34/36 (94.4%) vs. Solifenacin 27/36 (75.0%); *p* = 0.02. Complete symptom resolution: Mirabegron 8/36 (22.2%) vs. Solifenacin 3/36 (8.3%); *p* = 0.1	Mirabegron (*n* = 36): adverse effects 7/36 (19.4%) – headache 3/36 (8.3%), chest pain 1/36 (2.8%), others mild. Solifenacin (*n* = 36): adverse effects 17/36 (47.2%) – constipation 6/36 (16.7%), headache 6/36 (16.7%), skin rash 1/36 (2.8%), others mild	Mirabegron was more effective than solifenacin and had fewer adverse effects
Soliman et al., [16]	Prospective, randomized, single-blind, controlled trial	Children aged 5–14 years with newly diagnosed idiopathic OAB after at least 3 months of behavioral therapy	Mirabegron 50 mg once daily	Solifenacin 5 mg	Success rate (> 50% improvement in OAB symptoms): Mirabegron 56/64 (87.5%) vs. Solifenacin 58/65 (90.2%);*p* = 0.8. Complete dryness: Mirabegron 20/64 (31.3%) vs. Solifenacin 22/65 (33.8%);, *p* = 0.9	Mirabegron (*n* = 64): dry mouth 2 (2.8%), constipation 2 (2.8%), dizziness 2 (2.8%), abdominal cramps 2 (2.8%), blurred vision 1 (1.4%), nasopharyngitis 1 (1.4%), nausea 1 (1.4%), headache 1 (1.4%). Solifenacin (*n* = 65): dry mouth 7 (10.8%), constipation 8 (12.3%), dizziness 4 (6.2%), abdominal cramps 2 (3.1%), blurred vision 2 (3.1%), behavior change 2 (3.1%)	Both drugs are equally effective, but mirabegron had fewer and milder adverse effects

Notes: OAB = overactive bladder; DVSS = Dysfunctional Voiding Scoring System; SD = standard deviation

### Risk-of-bias assessment and publication bias

The observational study by Kim et al., evaluated with the NOS, scored 7 out of 9 stars, indicating good overall quality. It received the maximum score for selection, one star for comparability, and two stars for outcome assessment. Among the randomized controlled trials, Mansour et al. demonstrated a low overall risk of bias across all domains, with no concerns regarding randomization, deviations from intervention, missing outcome data, outcome measurement, or selective reporting. In contrast, Soliman et al. was judged to have a high overall risk of bias, primarily due to issues with the randomization process, deviations from the intended intervention, and selective reporting of outcomes, while outcome measurement and data completeness were considered low risk.

### Efficacy outcomes

**Fig. 2:**
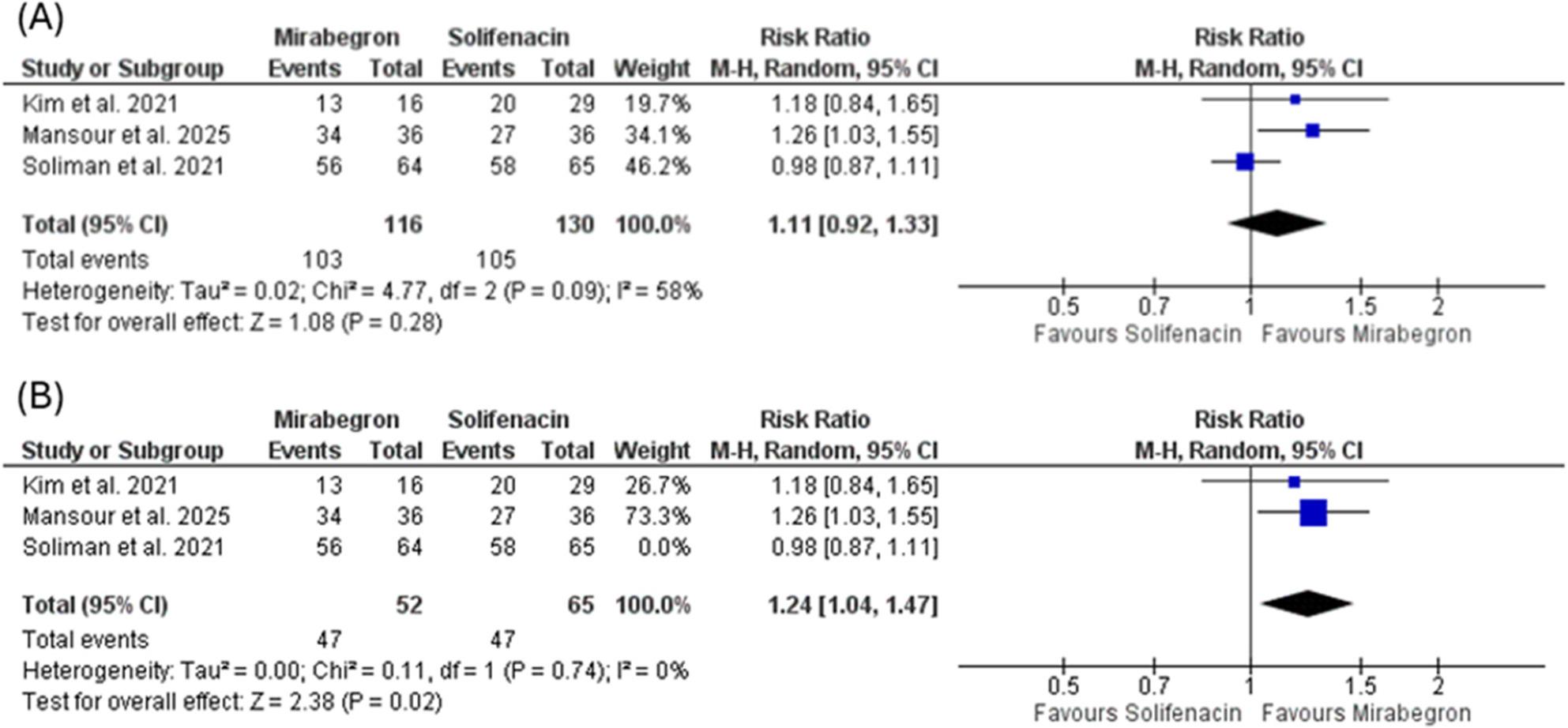
Forest plots of outcomes comparing mirabegron vs. solifenacin. **A** Forest plot of overall efficacy. **B** Forest plot of efficacy after excluding the study by Soliman et al. (2021) to resolve heterogeneity

The pooled analysis of all three studies indicated that mirabegron was not statistically superior to solifenacin in achieving treatment response (RR = 1.11, 95% CI: 0.92–1.33, *p* = 0.28). Notably, substantial heterogeneity was observed across studies (I² = 58%, *p* = 0.09), arising from differing treatment-response definitions. However, Explanatory sensitivity was done by excluding the study by Soliman et al. (assessed as high risk of bias), the pooled estimate from the remaining two studies demonstrated a statistically significant benefit of mirabegron over solifenacin (RR = 1.24, 95% CI: 1.04–1.47, *p* = 0.02), and in this sensitivity analysis heterogeneity was fully resolved (I² = 0%) Fig. 2.

### Safety outcome

**Fig. 3:**
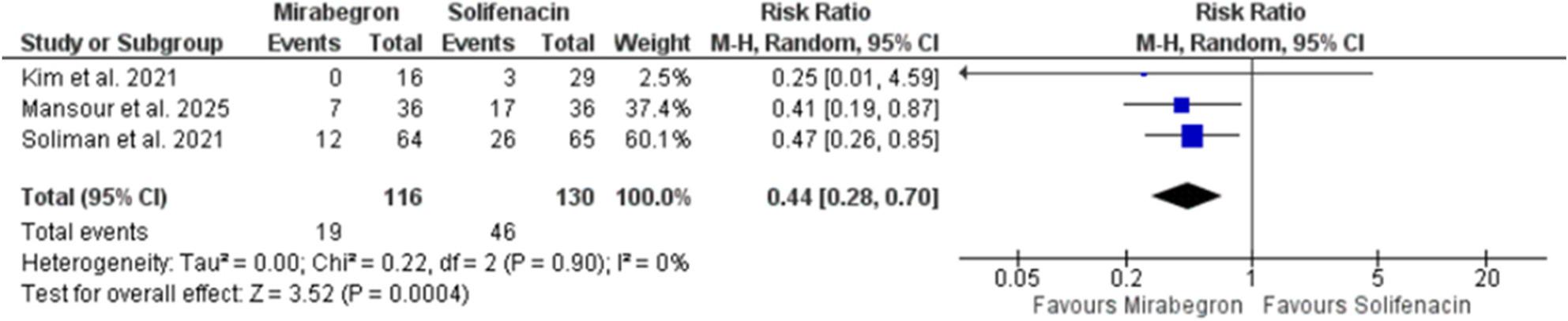
Forest plot of incidence of adverse events comparing mirabegron vs. solifenacin

As shown in Fig. 3, mirabegron was associated with a 56% lower risk of adverse events than solifenacin. The incidence of adverse events was 16.4% (19/116) for mirabegron and 35.4% (46/130) for solifenacin. The pooled risk ratio was 0.44 (95% CI 0.28–0.70; Z = 3.52, *p* = 0.0004), with negligible heterogeneity (I² = 0%). All three studies favoured mirabegron, with Soliman et al. and Mansour et al. contributing the most to the pooled effect.

## Discussion

This systematic review and meta-analysis summarized current evidence comparing the efficacy and safety of mirabegron and solifenacin in children with non-neurogenic OAB. Across three studies involving 246 patients, pooled analysis suggested no difference in treatment response. However, after excluding the high-risk study, the sensitivity analysis suggested that mirabegron had superior efficacy. This finding is based on only two studies with a combined sample size of 117 patients and should be interpreted cautiously as hypothesis-generating rather than confirmatory. Notably, mirabegron demonstrated a 56% lower risk of treatment-related adverse events, indicating a better short-term safety profile.

The studies included children ranging from 5 to 15 years of age (mean age 6–10 years), which is typical for pediatric OAB populations [2]. At baseline, symptom burden was similar across groups, with common OAB features such as urgency, frequency, daytime incontinence, and nocturnal enuresis [4, 27, 28]. Differences in study design may explain variations in reported efficacy. For example, Kim et al. defined response as ≥ 90% symptom improvement, while Mansour and Soliman defined response as ≥ 50% symptom improvement [16–18]. This variability highlights the need for standardized outcome measures in future pediatric OAB research.

Our results showed that mirabegron might be effective as solifenacin in children with OAB, which is consistent with findings in adults. Wang et al. reported no significant differences between mirabegron 50 mg and solifenacin 5 mg in reducing incontinence, urgency, or micturition frequency [29]. These results align with those of a trial conducted by Batista et al. and studies by Jamil et al. and Scaldazza et al., which reported comparable symptom relief [30–32]. Likewise, Soliman et al. and Kim et al. found no superiority of either drug in pediatric populations [16, 17]. Conversely, Mansour et al. reported higher response rates with mirabegron [18]. Solifenacin blocks M3 muscarinic receptors, while mirabegron stimulates β3-adrenergic receptors, but both drugs lead to detrusor muscle relaxation during bladder storage [9, 33, 34]. This shared physiological effect may explain their comparable efficacy in reducing urgency, frequency, and incontinence. However, variations in results across studies likely arise from differences in patient populations, baseline symptom severity, prior treatment exposure, and the tools used for symptom evaluation. However, given the limited number of included studies and small sample sizes, these findings should be considered preliminary. Larger, adequately powered pediatric RCTs are needed to determine whether any subtle differences in efficacy exist between mirabegron and solifenacin.

Beyond pharmacologic mechanisms and studies’ variability, a crucial clinical consideration is the overlap between OAB and dysfunctional voiding [35]. These conditions often coexist, with nearly half of patients with dysfunctional voiding requiring treatment for OAB, and vice versa [36]. Although studies in our meta-analysis excluded children with known dysfunctional voiding, diagnostic overlap could contribute to variable treatment responses and should prompt clinicians to evaluate concomitant dysfunctional voiding when OAB treatments yield incomplete responses.

In terms of safety, our analysis suggested that mirabegron was associated with a significantly lower incidence of adverse events than solifenacin in children, with overall event rates of 16.4% versus 35.4%, representing a relative reduction of approximately 56%. Adverse events associated with solifenacin were most commonly constipation, dry mouth, and headache, whereas mirabegron was associated with fewer and generally milder effects, such as headache or transient gastrointestinal discomfort. These results are consistent with previous studies in adults, where anticholinergic agents like solifenacin are consistently linked to constipation and dry mouth, while mirabegron demonstrates a more favourable safety profile [37–39]. This difference is likely due to their pharmacological mechanisms, as solifenacin has anticholinergic effects that affect multiple organs, whereas mirabegron acts as a β3-adrenergic receptor agonist, selectively targeting detrusor smooth muscle with limited systemic spillover or off-target effects that are common with solifenacin [9, 40]. The better tolerability of mirabegron is important in pediatrics, where side effects often cause treatment discontinuation [41]. By lowering this risk, mirabegron may support adherence and symptom control, potentially offering a safer short-term option for treating OAB in children within the limitations of current evidence.

Our findings related to non-neurogenic (idiopathic) OAB, for which neither mirabegron nor solifenacin has pediatric approval. Mirabegron received FDA approval in 2021 for neurogenic detrusor overactivity (NDO) in children ≥ 3 years, but remains off-label for idiopathic pediatric OAB [42]. Similarly, solifenacin has pediatric approval only for NDO [43]. Nevertheless, both agents are commonly prescribed in practice, highlighting the need for systematic evidence synthesis to guide decision-making without formal regulatory guidance.

### Current treatments and future perspectives

Antimuscarinic agents are the primary treatment for pediatric OAB, with oxybutynin and tolterodine being the only approved medications [7]. However, their limited uroselectivity and associated adverse effects contribute to poor treatment persistence (11.8%) and high discontinuation rates (32%) [1, 2]. Concerns have also been raised regarding neurodevelopmental safety, particularly because oxybutynin crosses the blood–brain barrier. Nonetheless, current evidence has not demonstrated measurable cognitive harm [44, 45]. However, long-term neurodevelopmental data remain limited.

Considering these limitations, β3-adrenoceptor agonists have emerged as alternative therapies [13]. Despite their therapeutic potential, cardiovascular safety remains a key concern, as Chapple et al. reported increases in heart rate and blood pressure, necessitating regular monitoring [12]. To optimize efficacy while mitigating dose-related adverse effects, dual therapy has been explored. In pediatric overactive bladder, this approach combines an antimuscarinic, typically solifenacin, with the β3-agonist mirabegron to enhance bladder relaxation through complementary mechanisms [46]. In adults, combination therapy has demonstrated greater efficacy than monotherapy [47–49]. Similarly, in children refractory to antimuscarinics, add-on mirabegron improved bladder capacity and continence with generally mild side effects [46, 50]. Overall, dual therapy appears promising and well-tolerated; however, randomized pediatric trials are still needed.

Beyond established therapies, Vibegron is a highly selective β3-adrenoceptor agonist with a potentially more favorable cardiovascular profile than mirabegron [51]. A comprehensive adult review confirmed vibegron’s high β3-AR selectivity, effective symptom relief, minimal cardiovascular effects, and favorable tolerability that may support better long-term adherence [51]. Kato et al. reported the first pediatric case of vibegron use for anticholinergic-resistant neurogenic detrusor overactivity, demonstrating improved bladder compliance and resolution of detrusor overactivity on urodynamic assessment [52]. Subsequently, Kitta et al. provided the first urodynamic evaluation of vibegron in children and adolescents with idiopathic OAB, documenting improvements in both subjective symptoms and lower urinary tract function [53]. These findings suggest that vibegron may be a promising option for children refractory to anticholinergics, although larger prospective studies are required to confirm its safety and long-term efficacy.

In parallel with pharmacologic therapies, high-dose vitamin D supplementation (VDS) has been explored as a safe and accessible strategy for pediatric OAB. In a three-arm randomized clinical trial, Chen et al. demonstrated that high-dose VDS (2400 IU/day) combined with standard urotherapy resulted in greater improvements in voiding frequency compared with solifenacin plus urotherapy or urotherapy alone, with an excellent safety profile [54]. A secondary analysis indicated that the therapeutic effect was more pronounced in children with baseline vitamin D levels of 20–35 ng/mL and more severe symptoms [55].Vitamin D deficiency was also more prevalent in children with OAB-related urinary incontinence than in healthy controls, and supplementation was shown to reduce urinary symptoms and improve quality of life in treatment-resistant cases [56]. These findings suggest that vitamin D may influence bladder function and that baseline vitamin D status could modify treatment response; however, larger studies are needed to confirm mechanisms, optimal dosing, and long-term outcomes.

When pharmacological therapy is ineffective or poorly tolerated, third-line therapies like intradetrusor botulinum toxin A (BoNT-A) injections and percutaneous tibial nerve stimulation (PTNS) are considered, though their pediatric use is limited by practical constraints [57–59]. BoNT-A is FDA-approved for pediatric neurogenic detrusor overactivity and benefits refractory OAB, with response rates of around 50% [58–60]. However, its effects last only 6–9 months, requiring repeat injections under anesthesia, increasing cost risks, procedure complications, and family burden [61]. PTNS has shown efficacy, with improved durability versus antimuscarinic therapy alone, but requires weekly 30-minute office sessions plus maintenance therapy, while pediatric in-home protocols remain unstudied [62, 63]. Although clinically effective, the requirement for repeated in-office treatments may limit accessibility and long-term adherence in children, highlighting the need for validated home-based neuromodulation strategies.

For this reason, focus has shifted toward noninvasive neuromodulation strategies. A systematic review and meta-analysis of eight randomized trials found that parasacral transcutaneous electrical nerve stimulation (TENS) produced 3.34-fold higher complete response rates than standard therapy, without significant adverse effects [64] Lin et al. reported that transcutaneous pelvic floor magnetic stimulation combined with voiding training achieved superior outcomes compared with voiding training alone in children with OAB [65]. Sacral neuromodulation (SNM) is being explored in refractory pediatric cases. Although sacral neuromodulation is not FDA-approved for children under 16 years for urological indications, off-label pediatric studies have demonstrated overall symptom improvement in approximately 68% of patients and complete resolution in 39%, with concurrent improvements in quality of life and urodynamic findings; however, these benefits must be weighed against notable complication and reoperation rates [66]. SNM may be considered a viable third-line option in carefully selected pediatric patients, while emphasizing the need for standardized protocols and larger multicenter studies to define long-term efficacy.

The findings of this meta-analysis should be considered preliminary due to several key limitations. Only three studies met the inclusion criteria, and with relatively small sample sizes, which limits the statistical power and generalizability of our findings. The substantial variability among the included studies in terms of design, follow-up duration, and definitions of treatment response constitutes a major methodological limitation. This inconsistency not only undermines confidence in the pooled efficacy estimate but also likely contributed significantly to the observed heterogeneity. Importantly, the safety conclusions of this meta-analysis are limited to short-term adverse events observed during follow-up periods of 12–34 weeks. Therefore, the long-term safety of mirabegron and solifenacin in the pediatric population cannot be determined based on the currently available evidence. Furthermore, none of the studies evaluated cognitive outcomes, which is an important factor when comparing antimuscarinic and non-antimuscarinic therapies in children [67]. Finally, because the included studies enrolled children aged 5–15 years, the findings may not be generalizable to infants, toddlers, or older adolescents.

Despite these limitations, our study is the first meta-analysis to compare mirabegron and solifenacin in the treatment of pediatric OAB directly. The results suggest that mirabegron may be an effective and safer alternative; however, further high-quality, randomized controlled trials with standardized outcome definitions and longer follow-up periods are needed.

## Conclusions

This systematic review and meta-analysis summarized evidence comparing mirabegron and solifenacin in pediatric OAB. The pooled data suggested comparable efficacy, with mirabegron showing a favorable short-term adverse event profile. However, given the few included studies and limited sample size, these findings are preliminary. Larger, multicenter RCTs with standardized outcomes and longer follow-up are needed to confirm these observations and inform clinical decisions.

## Supplementary Information

Supplementary Material 1. Appendix 1 is available in PDF format and contains the complete search strategies for the four databases, including all queries, keywords, and Boolean operators.

Supplementary Material 2. PRISMA_2020_checklist is available in PDF Format and contains the PRISMA Guidelines-reported items and their locations within the manuscript.

## Data Availability

All relevant data are available within the article and supplementary files.

## Abbreviations

AE: Adverse Event
CI: Confidence Interval
DVSS: Dysfunctional Voiding Scoring System
OAB: Overactive Bladder
RCT: Randomized Controlled Trial
RR: Risk Ratio
SD: Standard Deviation
