# Correction: Efficacy and safety of mirabegron versus solifenacin in the treatment of overactive bladder in children: a systematic review and meta-analysis

**DOI:** 10.1186/s12894-026-02179-1

**Published:** 2026-05-29

**Authors:** Mostafa Shaheen, Omar Mohamed Ali, Abdullah A. Draz, Amro K. Barakat, Ahmed Elghattas, Toqa Elbaz, Iyad Walid, Tasnim Tarboush, Asmaa Elsayed, Tasneem Mahmoud, Esraa Mohamed Elkhateeb, Abdallah Adel Nofal, Emad Samaan

**Affiliations:** 1 Faculty of Medicine, Mansoura University, Mansoura, 35516 Egypt; 2 Faculty of Medicine, Mansoura Manchester Research Society (MMRS), Mansoura University, Mansoura, Egypt; 3 Department of Internal Medicine – Nephrology Unit, Mansoura University Hospitals, Mansoura, Egypt

**Correction: BMC Urology 26, 100 (2026)**

**https://doi.org/10.1186/s12894-026-02155-9**

In the sentence beginning with “Randomized controlled trials were evaluated with.”, the current description of the Cochrane Risk of Bias tool version 2 (RoB 2) refers to seven domains. However, RoB 2 actually evaluates five domains in the Quality assessment and publication bias heading under Methods section of this article [1].

The corrected sentence is:

Randomized con­trolled trials were evaluated with the Cochrane Risk of Bias tool 2 (RoB 2) [22] which covers five domains: the randomization process, deviations from intended interventions, missing outcome data, measurement of the outcome, and selection of the reported result. Each domain is rated as ‘low risk,’ ‘high risk,’ or ‘some concerns.’

Also, Ref. 18 was incorrect and should have been.

Mansour I, Laymon M, Abdelhalim A, Dawaba MS, El-Hefnawy AS. Efficacy and Safety of Mirabegron Compared to Solifenacin in Treatment of Non-neurogenic Overactive Bladder in Children: A Randomized Controlled Trial. Int Braz J Urol. 2025;51. 10.1590/S1677-5538.IBJU.2024.0425.

The original article has been corrected.
