# Supplementary material for: Efficacy and safety of mirabegron versus solifenacin in the treatment of overactive bladder in children: a systematic review and meta-analysis
Source: BMC Urol. 2026 Apr 18;26:100. doi: 10.1186/s12894-026-02155-9 (PMC13101355; doi:10.1186/s12894-026-02155-9)
Supplement: Supplementary file 1 — Supplementary Material 1. Appendix 1 is available in PDF format and contains the complete search strategies for the four databases, including all queries, keywords, and Boolean operators. [file 12894_2026_2155_MOESM1_ESM.pdf]

Search strategy

("overactive bladder" OR OAB OR "overactive detrusor" OR "bladder overactivity" OR "detrusor overactivity" OR "urinary urgency" OR "urge incontinence" OR "urinary frequency" OR "lower urinary tract symptoms" OR LUTS) AND (mirabegron OR Betmiga OR Myrbetriq OR "beta3 adrenergic receptor agonist" OR "β3 adrenergic receptor agonist" OR "beta-3 adrenergic agonist" OR "β3-adrenoceptor agonist") AND (solifenacin OR Vesicare OR VESIcare OR "muscarinic antagonist" OR antimuscarinic OR "M3 receptor antagonist") AND (child OR children OR pediatric OR paediatric OR adolescent OR adolescence OR youth OR youths OR boy OR boys OR girl OR girls OR schoolchild* OR school-age OR teenager OR juveniles)
